# In vivo assessment of anti-helminthic and anti-inflammatory effects of Fucoidan on *Schistosoma mansoni* immature stages

**DOI:** 10.1038/s41598-026-56392-x

**Published:** 2026-06-17

**Authors:** Suzan H. Elgendy, Wafaa A. Aboukamar, Marwa M. Hamouda, Amira Ismail, Rehab T. Eldesoky, Ayat A. Elblihy

**Affiliations:** 1 Department of Medical Parasitology, Faculty of Medicine, Mansoura University, Mansoura, Egypt; 2 Department of Pathology, Faculty of Medicine, Mansoura University, Mansoura, Egypt

**Keywords:** Fucoidan, *Schistosoma mansoni*, Schistosomiasis, Granulomatous inflammation, Immunomodulation, Diseases, Immunology, Medical research, Microbiology

## Abstract

*Schistosoma mansoni* is a major cause of schistosomiasis, a neglected tropical disease that induces granulomatous inflammation, fibrosis, and liver damage. Fucoidan (FUC), a sulfated polysaccharide from brown seaweed, was evaluated for its anti-helminthic, anti-inflammatory, and antifibrotic effects against immature stages of *S. mansoni* in mice. Forty-eight CD-1 Swiss male albino mice were infected and allocated into six groups: infected untreated control, praziquantel-treated, and FUC-treated groups at 7, 21, 35, and 42 days post-infection. Treatment with FUC at 7, 21, and 35 dpi significantly reduced worm burden, granuloma size, fibrosis, and the expression of TNF-α, IL-1β, and iNOS in liver tissue. The strongest antipathological effects were observed with early-to-mid treatment, particularly FUC7, FUC21, and FUC35. In contrast, FUC42 showed weaker benefit, indicating that efficacy is timing-dependent. These findings suggest that FUC may be a promising candidate for early intervention in *S. mansoni* infection.

## Introduction

Schistosomiasis is a neglected tropical disease impacting over 250 million people globally, caused by *Schistosoma* blood flukes. The life cycle of this parasite requires both vertebrate hosts, including humans, and invertebrate hosts. Humans are infected when cercariae are released by the flukes, penetrate the skin and transform into schistosomula. These schistosomula then travel through the circulatory system, moving from the lungs to the liver’s blood vessels. About four weeks after infection, female worms start laying eggs that pass through the walls of the mesenteric vessels^1^.

A significant portion of the pathology and morbidity associated with schistosomiasis is linked to immune response-induced inflammation caused by eggs trapped in host tissues^2^. While it was previously thought that immune modulation primarily arose from eggs produced by adult worms, increasing evidence indicates that immune regulation begins at the point of infection and persists through all stages of the parasite’s lifecycle. Furthermore, *Schistosoma* parasites have developed strategies to evade the host’s immune defenses, including the modulation of cytokines to either promote or suppress inflammation and adjustments between Type 1 (Th1) and Type 2 (Th2) immune responses. Following infection with the parasite, the initial immune response in the infected tissues and plasma is characterized by type-1 inflammation, driven by IL-1, IL-12, tumor necrosis factor-α, and interferon-γ. During chronic schistosomiasis, this response is subsequently subdued by the CD4 + T helper 2 (Th2) response, which is triggered by antigens released by the eggs and mediated by IL-4, IL-5, IL-10, and IL-13^3^.

Schistosomiasis control in endemic regions has traditionally depended on mass drug administration of praziquantel (PZQ), which targets only adult worms. Despite its use, reinfections are frequent, and tissue damage often persists post-treatment. This reliance on a single drug raises concerns about potential drug-resistant parasites^4^, highlighting the urgent need for new treatments and vaccines 1,2.

**Fig. 1:**
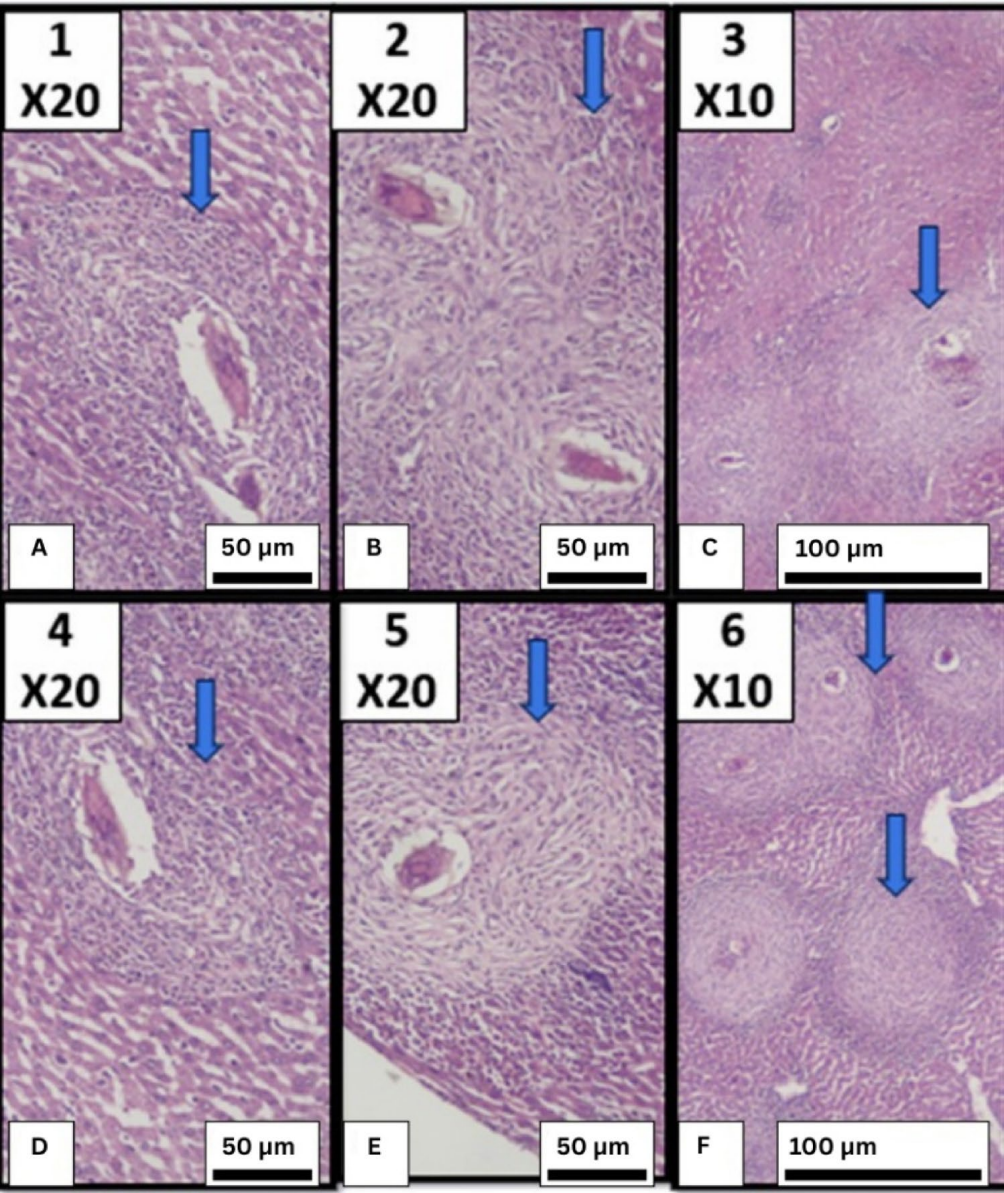
Photomicrographs of liver sections stained with Hx & E stain from infected untreated group (1 X20) and praziquantel treated group (2 X20) showed infiltration of the liver tissue by multiple bilharzial granulomas, some of which are fused to form large confluent ones; FUC7 treated group (3 X10); FUC21 treated group (4 X20) and FUC35 treated group (5 X20) showed focal infiltration of liver tissue by small sized granulomas without bilharzial ova or with degenerated bilharzial ova in most of them; FUC42 treated group, (6 X10) showed partial infiltration of the liver tissue by bilharzial granulomas. Some of which are centered around degenerated ova. Other sections show decrease in granulomas as regard size and number.

**Fig. 2:**
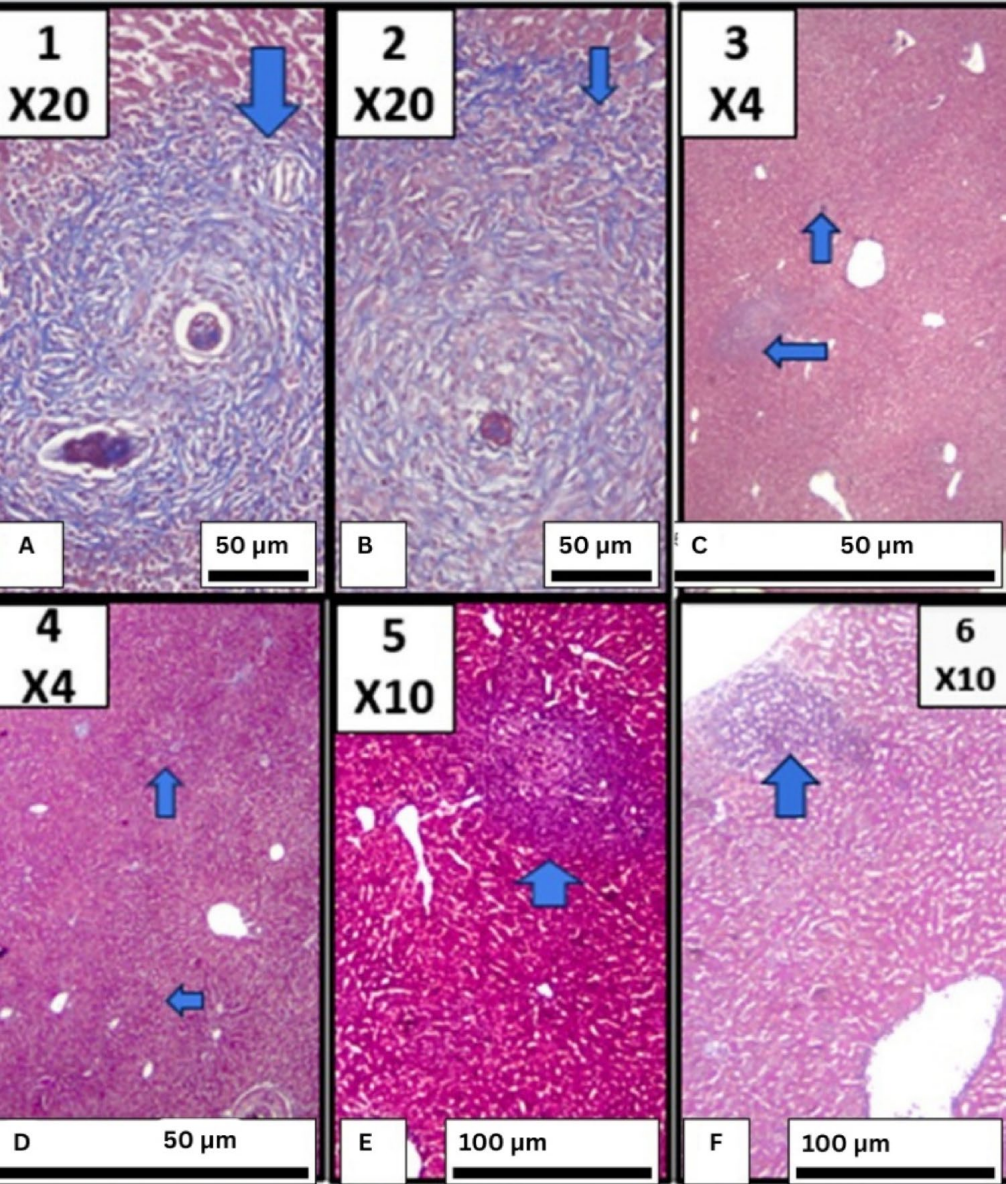
Photomicrographs of liver sections stained with Masson Trichrome from infected untreated group (1 X20) and praziquantel treated group (2 X20) showed that some of bilharzial granulomas are cellular, others are fibro-cellular centered around bilharzial ova; FUC7 treated group (3 X4); FUC21 treated group (4 X4); FUC35 treated group (5 X10) showed fibrocellular degenerated bilharzial granulomas; and FUC42 treated group (6 X10) showed fibrocellular granuloma.

Fucoidans (FUC), sulfated heteropolysaccharides from brown seaweeds (Phaeophyceae)^5^, offer antioxidant, immunomodulatory, anti-inflammatory, and anticancer benefits^6^. Fucoidans regulate the inflammatory reaction, inhibited inflammatory enzymes, cyclooxygenase-2, interleukin 1β and matrix metallopeptidase 9^7^. Fucoidan has been found to possess a potent antioxidant activity against free radicals such as hydrogen peroxide, superoxide anion, singlet oxygen and nitric oxide thus preventing variant oxidative stress-related diseases due to their fucose backbone^8^.

Recently combined FUC and PZQ treatment revealed a potential antifibrotic effect of fucoidan on the liver tissue and expressed marked improvement of schistosome-induced pathology, hence, FUC could be considered as a promising adjuvant therapy to PZQ to induce PZQ efficiency and alleviate the associated fibrotic lesions^9^ 3,4

**Fig. 3:**
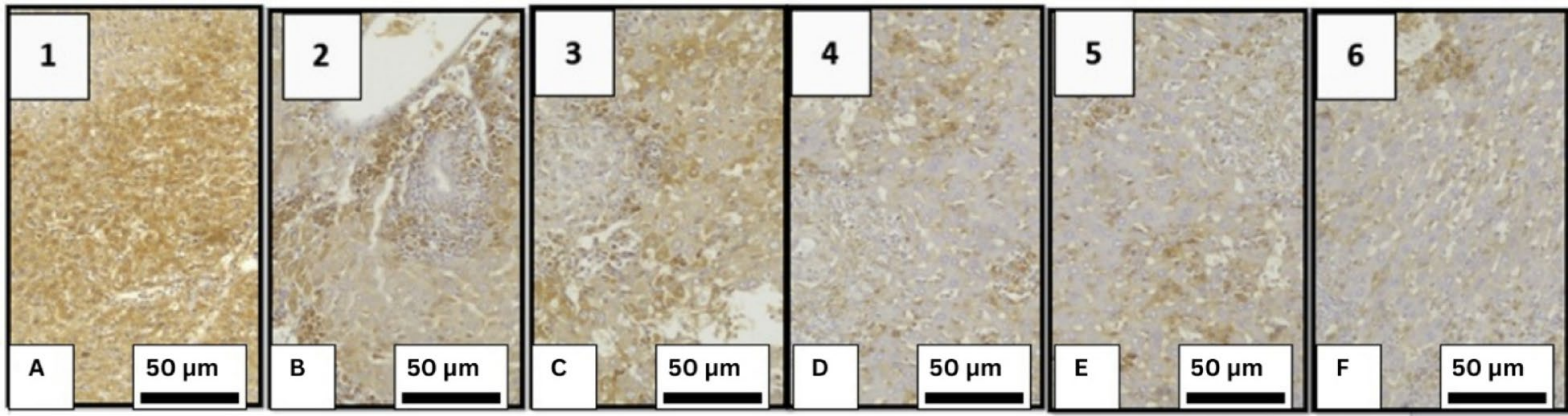
Photomicrographs of liver sections stained with TNF (X20) from infected untreated group (1) showed diffuse cytoplasmic reaction. Praziquantel treated group (2) and FUC7 treated group (3) showed partial staining of hepatocytes and inflammatory cells. FUC21 treated group (4 × 20); FUC35 treated group (5 × 20) and FUC42 treated group (6 × 20) showed only focal staining of both hepatocytes and inflammatory cells (arrows).

**Fig. 4:**
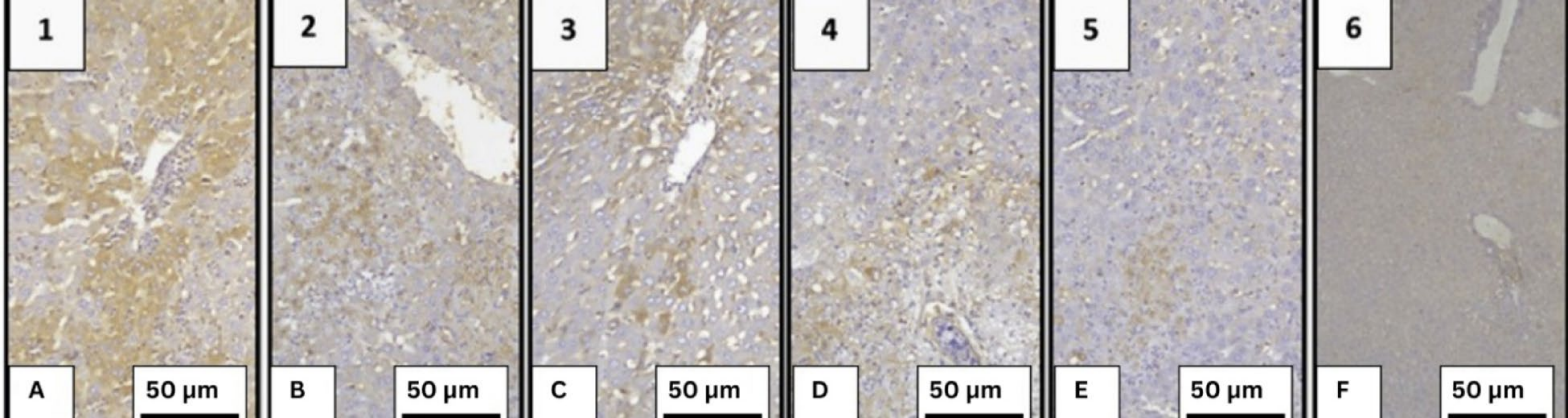
Photomicrographs of liver sections stained with IL1 (X20) from infected untreated group (1) showed near diffuse cytoplasmic reaction of hepatocytes and inflammatory cells. Other groups show reduction of cells with positive reaction as regards percentage and intensity.

With regard to its toxicity, the 50% lethal dose in mice after single oral dose of FUC was estimated over 2000 mg/kg in both female and male mice. So, FUC is safe in our model tested.

We hypothesize that FUC might have a potential anti-Schistosomal effect mostly on juveniles concerning its anti-inflammatory, immunomodulatory, antioxidant and antifibrotic properties over praziquantel drug. Therefore, this study evaluated FUC therapeutic effects against *Schistosoma mansoni* in murine models at different infection timing with regard to its parasitological and histopathological aspects. As well as outline the recent findings that are related to the anti-inflammatory, immunomodulatory and antioxidant properties of FUC and their potential as novel molecules for the treatment of *Schistosoma mansoni*.

## Results

### Effect of drugs administration regarding parasitological parameters

Mice body weight and liver weight increased in the treated groups compared with the infected control, with a significant increase in body weight observed only in the PZQ-treated group. Total adult worm burden was significantly reduced in all treated groups compared with the infected control. PZQ achieved complete elimination of adult worms, whereas FUC produced partial but significant reductions, with greater benefit when administered earlier after infection. Liver egg burden was also significantly reduced in all treated groups, with the lowest egg count observed in the PZQ group, followed by FUC35 and FUC42^5^.

**Fig. 5:**
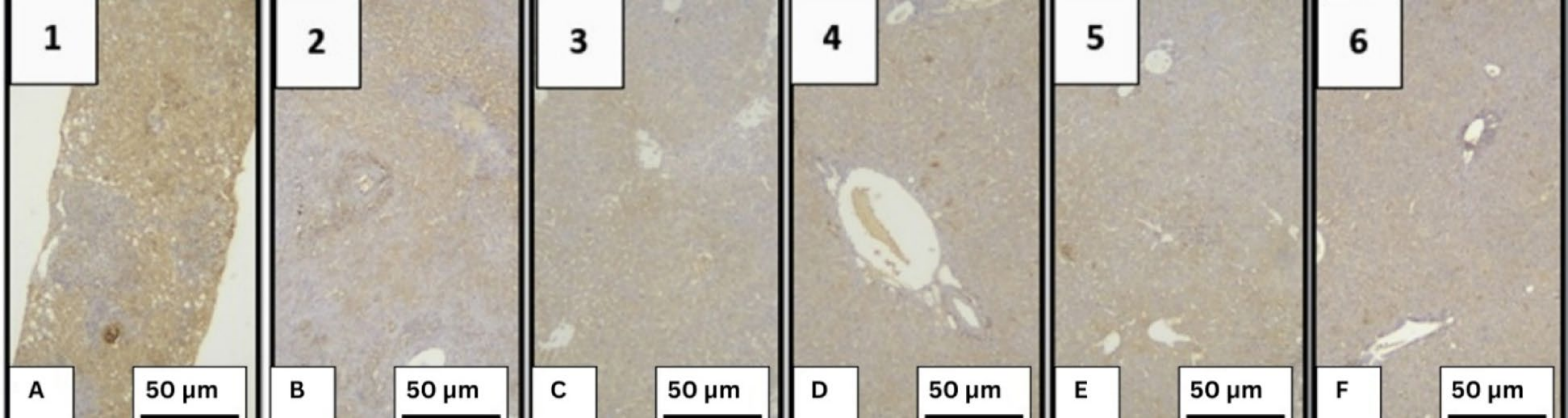
Photomicrographs of liver sections stained with iNOS (X20) from infected untreated group (1) showed near diffuse cytoplasmic reaction of hepatocytes. Other groups showed reduction of cells with positive reaction as regards percentage and intensity.

The proportion of immature eggs differed significantly among groups (P = 0.01), with FUC42 showing the highest immature egg percentage. By contrast, FUC7, FUC21, and FUC35 maintained higher proportions of mature viable eggs (Table 1).

**Table 1:** Effects of administration of FUC and PZQ on *S. mansoni*-infected mice regarding body weight, liver weight, adult count, egg load, mature egg percentage and immature egg percentage.

Animal groups	Mice weight (gm)	Liver weight (gm)	Adult count	Egg load	Mature egg (%)	Immature egg (%)
	(Mean ± SD)	(Mean ± SD)	Median (min–max)	Median (min–max)	Median (min–max)	Median (min–max)
Group 1 (Control group)	22.8 ± 4.1	1.8 ± 0.29	21 (6–32)	14,000 (3500–15,000)	40 (40–90)	60 (10–60)
Group 2 (PZQ group)	31 ± 7.7 ^a^	2.1 ± 0.22	0 (0–0)^a^	750 (500–1000)^a^	25 (0–50)	75 (50–100)
Group 3 (FUC7 group)	29.3 ± 1.1	2.1 ± 0.19	11 (3–13) ^b^	3500 (3000–4000)^ab^	60 (50–70) ^bf^	40 (30–50)^b^
Group 4 (FUC21 group)	28 ± 3.1	2.1 ± 0.14	3 (3–13)^ab^	2500 (500–2500) ^ab^	60 (30–70) ^bf^	40 (30–70) ^b^
Group 5 (FUC35 group)	30 ± 2.2	2.1 ± 0.27	10 (7–13)^b^	1000 (1000–1000) ^ac^	60 (50–70) ^bf^	40 (30–50) ^b^
Group 6 (FUC42 group)	26.1 ± 3.8	2.1 ± 0.16	7 (6–15) ^b^	2000 (2000–3000) ^abce^	10 (5–100)	90 (30–95) ^acde^
Test of significance	F = 2.9 P = 0.03*	F = 9.1 P ≤ 0.001*	KW = 20.9 P = 0.001*	KW = 28.7 P ≤ 0.001*	KW = 9.7 P = 0.04*	KW = 14.2 P = 0.01*

Data expressed as mean ± SD or median (minimum–maximum).

*Significant p ≤ 0.05.

F: One way ANOVA test.

KW: Kruskal–Wallis Test.

^a^P < 0.05 vs control group, ^b^ P < 0.05 vs PZQ group, ^c^ P < 0.05 vs FUC7 group, ^d^ P < 0.05 vs FUC21 group, ^e^ P < 0.05 vs FUC35 group, ^f^ P < 0.05 vs FUC42 group.

Significant differences between groups were done by Post Hoc Tukey test when F is significant or by Mann Whitney test if KW is significant.

### Effect of drugs administration regarding histopathological parameters

*Schistosoma* granulomas were evaluated in liver tissue and portal areas with respect to type, stage, size, inflammatory cell infiltration, focal necrosis around the central vein, fibrosis, and the proportion of intact and degenerated ova. Granuloma composition and stage varied with treatment timing. Early FUC treatment shifted granulomas toward the cellular and stage 1 pattern, whereas later treatment groups showed more fibrocellular and stage 3 lesions.

Liver specimens from FUC7-, FUC21-, and FUC35-treated mice showed predominantly cellular granulomas, whereas fibrocellular granulomas were more common in the infected control, praziquantel-treated, and FUC42-treated groups. In contrast, FUC42-treated mice mainly exhibited fibrocellular granulomas.

Inflammatory cell infiltration and focal necrosis around the central vein also differed among groups. Marked infiltration was observed in the infected control group (60%), moderate infiltration in the PZQ and FUC42 groups (66.7% and 57.1%, respectively), mild infiltration in the FUC7 group (66.7%), and minimal infiltration in the FUC21 and FUC35 groups (66.7% and 83.3%, respectively), with a significant difference among groups.

Granuloma fibrosis was significantly decreased in FUC7, FUC21, FUC35 (8.3%), FUC42 (44.3%), and PZQ (43.3%) compared with the infected control (68%). However, fibrosis reduction was markedly greater in the FUC7, FUC21, and FUC35 groups than in the PZQ and FUC42 groups. Most granulomas in the FUC7 (50%), FUC21 (60%), and FUC35 (60%) groups contained degenerated ova, which differed significantly from the infected control, PZQ-treated, and FUC42-treated groups, where granulomas mainly contained intact ova (98%, 95.8%, and 93.6%, respectively).

Granuloma size also decreased significantly after FUC7, FUC21, and FUC35 treatment, reaching approximately half the diameter observed in infected controls. By comparison, praziquantel and FUC42 produced only modest size reductions (Figure1, Figure 2, Table 2 and 3).

**Table 2:** Effects of administration of Fucoidan (FUC) and PZQ on *S. mansoni*-infected mice regarding inflammatory cell infiltrate, focal necrosis around the central vein and granuloma type.

Animal groups	Inflammatory cell infiltrate/ Focal necrosis around central vein		Granuloma type	
	Degree	N (%)	Types	N (%)
Group 1 Control group (n = 5)	Minimal Mild Moderate Marked	0 0 2 (40%) 3 (60%)	Fibrocellular Fibrous Cellular	4 (80%) 0 1 (20%)
Group 2 PZQ group (n = 6)	Minimal Mild Moderate Marked	0 0 4 (66.7%) 2 (33.3%)	Fibrocellular Fibrous Cellular	4 (66.7%) 2 (33.3%) 0
Group 3 FUC7 group (n = 6)	Minimal Mild Moderate Marked	2 (33.3%) 4 (66.7%) 0 0	Fibrocellular Fibrous Cellular	1 (16.7%) 0 5 (83.3%)
Group 4 FUC21 group (n = 6)	Minimal Mild Moderate Marked	4 (66.7%) 2 (33.3%) 0 0	Fibrocellular Fibrous Cellular	1 (16.7%) 0 5 (83.3%)
Group 5 FUC35 group (n = 6)	Minimal Mild Moderate Marked	5 (83.3%) 1 (16.7%) 0 0	Fibrocellular Fibrous Cellular	1 (16.7%) 0 5 (83.3%)
Group 6 FUC42 group (n = 7)	Minimal Mild Moderate Marked	0 0 4 (57.1%) 3 (42.9%)	Fibrocellular Fibrous Cellular	5 (71.4%) 2 (28.6%) 0
Test of significance	MC = 44.1 P < 0.001*		MC = 26.1 P = 0.002*	

Data expressed as number (%).

*significant p ≤ 0.05.

MC, Monte Carlo test.

**Table 3:** Effects of administration of Fucoidan (FUC) and PZQ on *Schistosoma mansoni-*infected mice regarding fibrosis in granuloma, granuloma stage, intact ova, degenerated ova and granuloma size:

Animal groups	Fibrosis in granuloma (%)	Stage of granuloma	Intact ova (%)	Degenerated ova (%)	Granuloma size (um)
	(Mean ± SD)	Median (min–max)	(Mean ± SD)	Median (min–max)	(Mean ± SD)
Group 1 Control group (n = 5)	68 ± 8.4 ^bcdef^	3 (3–4)	98 ± 2.7	0 (0–5)	1020 ± 83.7
Group 2 PZQ group (n = 6)	43.3 ± 15.1 ^acde^	3 (2–3)	95.8 ± 3.8	5 (0–10)	866.7 ± 51.6^a^
Group 3 FUC7 group (n = 6)	8.3 ± 2.6 ^abf^	1 (1–1) ^abf^	48.3 ± 7.5^ab^	50 (40–60) ^abf^	516.7 ± 75.3^ab^
Group 4 FUC21 group (n = 6)	8.3 ± 2.0 ^abf^	1 (1–1) ^abf^	40 ± 6.3^ab^	60 (50–70) ^abcf^	466.7 ± 51.6^ab^
Group 5 FUC35 group (n = 6)	8.3 ± 1.8 ^abf^	1 (1–1) ^abf^	40 ± 6.3^ab^	60 (50–70) ^abcf^	466.7 ± 40.5^ab^
Group 6 FUC42 group (n = 7)	44.3 ± 11.3 ^acde^	3 (2–3)	93.6 ± 4.8 ^cde^	10 (0–10)	828.6 ± 48.8 ^acde^
Test of significance	F = 47.7 P ≤ 0.001*	KW = 32.4 P ≤ 0.001*	F = 165.7 P ≤ 0.001*	KW = 29.6 P ≤ 0.001*	F = 89.2 P ≤ 0.001*

Data expressed as mean ± SD or median (minimum–maximum).

*significant p ≤ 0.05.

F: One Way ANOVA test.

KW: Kruskal–Wallis Test.

^a^P < 0.05 vs control group, ^b^ P < 0.05 vs PZQ group, ^c^ P < 0.05 vs FUC7 group, ^d^ P < 0.05 vs FUC21 group, ^e^ P < 0.05 vs FUC35 group, ^f^ P < 0.05 vs FUC42 group.

Significant differences between groups were done by Post Hoc Tukey test when F is significant or by Mann Whitney test if KW is significant).

### Effect of drugs administration regarding immunohistochemical staining

Administration of FUC at 7, 21, and 35 dpi significantly reduced TNF-α, IL-1β, and iNOS expression in liver tissue sections compared with the infected control, PZQ, and FUC42 groups. The reduction was weaker in FUC42, indicating that the anti-inflammatory effect depends on treatment timing (Figure3, Figure 4, Figure 5, Table 4).

**Table 4:** Effects of administration of FUC and PZQ on *S. mansoni*-infected mice regarding TNF-α, IL-1β, and iNOS immunostaining:

Animal groups	TNF	IL-1β	iNOS
	Median (min–max)	Median (min–max)	Median (min–max)
Group 1 Control group	7 (7–8)	7 (7–8)	3 (2–3)
Group 2 PZQ group	7 (6–7)	7 (6–7)	3 (2–3)
Group 3 FUC7 group	3 (2–4) ^abf^	3 (2–4) ^abf^	1 (1–2) ^abf^
Group 4 FUC21 group	2 (2–3) ^abf^	2 (2–3) ^abf^	1 (1–2) ^abf^
Group 5 FUC35 group	2 (2–3) ^abf^	2 (2–3) ^abf^	1 (1–1) ^abf^
Group 6 FUC42 group	6 (6–7)	6 (6–7)	3 (2–3)
Test of significance	KW = 30.3 P < 0.001*	KW = 30.3 P ≤ 0.001*	KW = 27.4 P ≤ 0.001*

Data expressed as mean ± SD or median (minimum–maximum).

*Significant p ≤ 0.05.

F: One Way ANOVA test.

KW: Kruskal–Wallis Test.

Pairwise comparisons are presented as superscript letters: ^a^ P < 0.05 vs control group, ^b^ P < 0.05 vs PZQ group, ^c^ P < 0.05 vs FUC7 group, ^d^ P < 0.05 vs FUC21 group, ^e^ P < 0.05 vs FUC35 group, ^f^ P < 0.05 vs FUC42 group.

Significant differences between groups were done by Post Hoc Tukey test when F is significant or by Mann Whitney test if KW is significant.

TNF-α expression decreased from a median score of 7 in the infected control group to 3, 2, and 2 in the FUC7, FUC21, and FUC35 groups, respectively, whereas it remained approximately unchanged after PZQ and FUC42 treatment (median scores of 7 and 6, respectively). IL-1β showed a similar pattern, with median scores of 3, 2, and 2 in the FUC7, FUC21, and FUC35 groups, respectively, compared with 7, 7, and 6 in the infected control, PZQ, and FUC42 groups. iNOS expression was also lower in the FUC7, FUC21, and FUC35 groups (median score 1) than in the infected control, PZQ, and FUC42 groups (median score 3).

## Discussion

This study provides a comprehensive evaluation of the therapeutic potential of fucoidan (FUC), a naturally occurring sulfated polysaccharide derived from brown seaweed (Fucus vesiculosus), against experimental infection with S. mansoni immature stages using parasitological, histopathological, and immunohistochemical approaches^5^. PZQ remains the mainstay of schistosomiasis treatment; however, its limited efficacy against juvenile schistosomula and concerns regarding reduced effectiveness in high-transmission settings underscore the need for alternative or adjunctive therapies^10,11^. Fucoidan has attracted interest because of its antioxidant, immunomodulatory, and anti-inflammatory properties, which may be relevant to parasite control and attenuation of infection-related tissue damage^5,6^.

In the present study, FUC produced time-dependent therapeutic effects. Body and liver weights increased in the treated groups compared with the infected controls, suggesting partial recovery of nutritional status and hepatic condition despite ongoing infection.

Total adult worm burden was significantly reduced in all FUC-treated groups; however, PZQ achieved complete elimination of adult worms, confirming its superior adulticidal activity. These findings are consistent with previous reports showing that fucoidan can reduce worm burden in schistosomiasis through indirect immunomodulatory effects rather than direct parasiticidal activity alone. They also agree with Mohamed et al., who reported a reduction in total worm burden in both PZQ- and FUC-treated mice, with greater reduction in the PZQ group^12^. In agreement with our findings, Mohamed et al.^9^ revealed that the percentage of total worm burden reduction in the PZQ treated group was 73.8% and with the FUC treated group by a reduction rate of 58.9%, compared with the infected untreated mice group.

The reduction in worm burden observed with FUC may reflect, at least in part, its capacity to modulate oxidative stress and host inflammatory responses, as previously suggested for other parasitic systems such as *Leishmania donovani*^13^. Similar anti-parasitic effects have also been reported for other seaweed-derived compounds, including Dictyota, which reduced adult *S. mansoni* counts in culture^14^. Together, these data support the notion that FUC exerts a moderate but meaningful anti-schistosomal effect that is most evident when administered early after infection.

Hepatic egg burden was markedly reduced following treatment, with the strongest reduction observed in the PZQ group, followed by FUC35 and FUC42. Importantly, FUC42 was associated with the highest proportion of immature eggs, whereas FUC7, FUC21, and FUC35 retained higher proportions of mature viable eggs. These findings indicate that the effect of FUC depends on treatment timing and is not uniform across all stages of egg development. Instead, the data supports a stage-dependent response, with treatment at later infection stages producing greater changes in egg viability and burden^15,16^. This interpretation is consistent with Ibrahim et al.^17^, who reported altered egg viability patterns in *S. mansoni* after treatment with pentoxifylline and PZQ.

Because egg viability is closely linked to granulomatous inflammation and transmission potential, these changes are biologically and clinically important. Previous work has suggested that the effect of FUC on egg production may also involve modulation of gut microbiota. Cortes et al. reported that *S. mansoni* infection reduces alpha diversity and depletes beneficial bacteria such as *Lactobacillus*^18^, whereas FUC may partially restore this dysbiosis by promoting *Lactobacillus* growth^19^. Because *Lactobacillus* has been associated with lower worm and egg burdens, this mechanism may contribute to the biological effects observed here^20^.

In addition, Mohamed et al.^9^ previously reported that FUC treatment at 60 dpi reduced worm burden, egg count, and immature ova, while increasing dead ova, supporting the broader anti-schistosomal potential of fucoidan.

Histopathological examination demonstrated pronounced timing-dependent differences in granuloma morphology and composition. Early FUC treatment (FUC7, FUC21, and FUC35) was associated with predominantly cellular granulomas, whereas infected controls, PZQ-treated mice, and FUC42-treated mice showed mainly fibrocellular granulomas. This shift toward a cellular pattern in the early treatment groups suggests that FUC may help limit progression toward more fibrogenic lesions^21^.

This pattern is consistent with the natural evolution of schistosomal granulomas, which initially contain predominantly inflammatory cells and later become more fibrotic as collagen deposition increases^16^.

It is also compatible with the idea that regulatory immune mechanisms emerge over time to restrain granuloma expansion. In this context, the benefit of early FUC administration may lie in preserving a less fibrogenic granuloma phenotype before chronic fibrotic remodeling becomes established^22^.

The granuloma stage data further support this interpretation, as FUC7, FUC21, and FUC35 predominantly exhibited stage 1 granulomas, whereas the infected control, PZQ, and FUC42 groups mainly showed stage 3 lesions. This stage distribution indicates that early FUC administration may prevent progression to advanced granulomatous stages characterized by extensive fibrosis and architectural disruption^23^.

This is further supported by the marked reduction in granuloma fibrosis in the early-to-mid treatment groups, which is particularly important because hepatic fibrosis is a major determinant of morbidity in chronic schistosomiasis^24^.

Inflammatory cell infiltration and focal necrosis around the central vein also showed a clear timing-dependent gradient. The infected controls exhibited marked inflammatory infiltration, whereas the PZQ and FUC42 groups showed moderate infiltration. In contrast, FUC7-treated mice displayed mild infiltration, and FUC21 and FUC35 groups showed minimal infiltration. These findings suggest that earlier FUC administration interrupts inflammatory cascades before more extensive tissue injury occurs^25,26^.

The reduction in granuloma size observed in FUC7, FUC21, and FUC35 further supports a morphometric benefit of early intervention, whereas PZQ and FUC42 produced only modest size reductions. Similar observations have been reported in S. japonicum infection, where FUC reduced granuloma size and fibrosis through suppression of pro-inflammatory signaling and enhancement of regulatory immune responses^12^.

The proportion of degenerated ova was significantly higher in FUC7, FUC21, and FUC35 groups than in the infected control, PZQ, and FUC42 groups. This suggests that early-to-mid FUC treatment may enhance egg degeneration by preserving an inflammatory environment capable of damaging entrapped ova before immune tolerance becomes dominant^17,27^. Since viable eggs continue to release immunostimulatory molecules that sustain granulomatous inflammation, increased egg degeneration is a biologically meaningful endpoint^16^.

Immunohistochemical analysis showed that FUC7, FUC21, and FUC35 significantly reduced hepatic TNF-α, IL-1β, and iNOS expression relative to the infected control, PZQ, and FUC42 groups. The weaker effect of FUC42 again supports a timing-dependent response. Reduced TNF-α expression may reflect suppression of NF-κB and MAPK-related inflammatory signaling, which has been described in multiple inflammatory models^28–30^. However, because TNF-α also contributes to granuloma formation and parasite control, these results should be interpreted as evidence of immunomodulation rather than simple anti-inflammatory suppression^26^.

Previous work by Mohamed et al.^9^ showed that combined FUC and PZQ treatment produced the greatest reduction in TGF-β expression compared with either monotherapy, supporting the relevance of this pro-fibrotic pathway in schistosomal pathology. Likewise, Abaza et al.^31^ documented significant immunomodulatory activity in experimentally infected *S. mansoni* mice, with decreased hepatic phosphorylated p65 and pro-inflammatory cytokines (IL-6, IL-12, and TNF-α), together with increased anti-inflammatory cytokines (IL-4 and IL-13) in FUC-treated groups.

IL-1β followed the same pattern as TNF-α. IL-1β is a key pro-inflammatory mediator that amplifies acute-phase responses and promotes cellular recruitment to granulomatous lesions^26^; therefore, its downregulation likely contributed to the reduced inflammatory pathology observed in this study. Fucoidan-mediated suppression of IL-1β may be mechanistically relevant because IL-1β is regulated by upstream inflammatory pathways, including NF-κB and MAPK signaling^32,33^.

Consistent with our findings, Bai et al.^12^ reported that FUC reduced hepatic inflammatory cytokine expression and phospho-p65 levels in S. japonicum-infected mice, supporting a similar anti-inflammatory mechanism in schistosomiasis.

iNOS expression also declined in the early-to-mid FUC groups. Although iNOS-derived nitric oxide can contribute to parasite killing, excessive production may promote oxidative stress and hepatocellular damage. Thus, the reduction observed here may reflect a more balanced host response, in which parasite control is maintained while tissue injury is limited. This interpretation is in line with previous reports that fucoidan can inhibit iNOS expression through NF-κB-related pathways in inflammatory settings^33,34^. In schistosomiasis, iNOS is induced primarily by inflammatory cytokines such as TNF-α and IL-1β, so the parallel reduction of these markers is biologically coherent^35,36^.

The present study has several important implications. First, it demonstrates that the therapeutic activity of fucoidan is strongly dependent on the timing of administration, with earlier treatment producing superior parasitological, histological, and immunohistochemical outcomes. Second, it suggests that FUC may be more useful as an adjunct or early-stage intervention than as a replacement for PZQ, which remains superior for complete adult worm elimination. Third, the data support a model in which fucoidan modulates host inflammatory and fibrotic responses rather than acting solely as a direct antiparasitic agent.At the same time, some limitations should be acknowledged. This study did not evaluate dose–response relationships, sex-related differences, or upstream signaling pathways such as NF-κB and TGF-β. In addition, the use of a single dose and a specific experimental model may limit generalizability. Further studies are warranted to determine the optimal combination regimen of PZQ and FUC and to explore its potential clinical relevance in acute schistosomiasis.

## Conclusion

In summary, this study demonstrates that FUC is a promising therapeutic candidate against *S. mansoni* infection, with efficacy strongly dependent on the timing of administration. Early-to-mid treatment with FUC significantly reduced worm burden and improved schistosomiasis-induced pathology by shifting granuloma phenotype toward a less fibrotic stage, reducing granuloma size and fibrosis, and suppressing pro-inflammatory mediators. Although PZQ remains superior for adult worm elimination, FUC showed greater benefit in limiting granuloma-related pathology, suggesting potential value as an adjunct or early-stage therapeutic candidate.

## Materials and methods

### Animals and infection

Forty-eight CD-1 Swiss male albino mice, weighing 20–22 g, were purchased from the Schistosome Biology Supply Center (Theodor Bilharz Research Institute, Giza, Egypt). Each mouse was infected with 50–60 *Schistosoma mansoni* cercariae by subcutaneous injection in 100 μL dechlorinated water, following previously described methods^37^. The mice were maintained in the laboratory of the Medical Parasitology Department, Mansoura Faculty of Medicine (air-conditioned at 20 ± 3 °C) and were provided with commercial standard pelleted diet and water ad libitum. All experimental procedures and animal handling protocols were reviewed and approved by the Animal Care and Use Committee (ACUC) of Mansoura University, Egypt (Approval Code: MU-ACUC [MED.R.24.09.44]; Approval Date: 15 September 2024), in accordance with institutional and national guidelines for the care and use of laboratory animals.

### Drugs

The following drugs were purchased from their manufacturing companies: Praziquantel® from Sedico Pharmaceutical Co. (6th of October City, Egypt) and 10% Dimethyl sulfoxide (DMSO) from Sigma-Aldrich (St. Louis, MO, USA) as a vehicle for dissolving PZQ. Fucoidan (from Fucus vesiculosus, Synergy co., Utah, USA) was dissolved in phosphate-buffered saline (PBS).

### Experimental design and drug administration

Six groups of mice (8 mice each group) were included in this study and allocated as: Group 1 Infected control mice that were left without treatment; Group 2 Infected treated control mice (PZQ) that received an oral dose of PZQ (500 mg/kg), divided into two successive days^38^, starting 42 dpi; Group 3 Infected treated mice (FUC7) that received an oral dose of FUC (500 mg/kg) for two successive days^12,30^, starting 7 dpi; Group 4 Infected mice (FUC21) that received an oral dose of FUC (500 mg/kg) for two successive days starting 21 dpi; Group 5 Infected treated mice (FUC35) that received an oral dose of FUC (500 mg/kg) for two successive days starting 35 dpi; Group 6 Infected treated mice (FUC42) that received an oral dose of FUC (500 mg/kg) for two successive days starting 42 dpi.

All drug administrations were performed via oral gavage. Mice of all groups were sacrificed in the ninth week post-infection by thiopental (100 mg/kg IP injection).

### Parasitological parameters

Adult *Schistosoma mansoni* worms were collected by perfusions of porto-mesenteric veins of infected groups according to the method described^39^. The perfused worms were counted using an inverted microscope. The adult worms were rinsed in PBS and fixed in 2.5% glutaraldehyde for 60 min at 4 °C for subsequent scanning electron microscopy.

The livers from all mice were dissected, weighed, and processed for parasitological and histopathological examination. Eggs located in the tissue of the liver (eggs per tissue gram) were assessed after dissolution in 0.5% KOH solution^40^.

For oogram assessment, parts of the intestine (about one cm long) were examined microscopically to determine the developmental egg stages according to Pellegrino et al.^41^. The eggs were classified based on their developmental stages as immature, mature, and dead eggs. Immature eggs were categorized according to the size of the developing embryo relative to the egg diameter, while mature eggs contained fully developed miracidia. Dead eggs were identified by their appearance as darkened, granular, semitransparent, or with retracted embryos.

### Histopathological assessment

Liver samples from all mice groups were fixed in neutral buffered formalin (10%) and processed to paraffin blocks. From each liver, Sects. (4 μm thickness) were prepared using a rotary microtome. The granuloma size was measured using a calibrated ocular micrometer, and the mean diameter of each granuloma was calculated by measuring two diameters at 90-degree angles to each other. Eggs morphology was evaluated according to the criteria described by Lichtenberg^42^.

Masson-trichrome staining of the liver sections was performed to examine the deposition of collagen, and the evolutionary stages of granulomas were classified according to Amaral et al.^43^. Granuloma stage was classified according to established histological criteria, and fibrosis was quantified as mean ± SD.Two histological sections from each sample were examined, and five random fields from each section were analyzed to ensure representative assessment of the tissue changes. The degree of fibrosis in the granulomas was quantified and expressed as mean ± standard deviation.

### Immunohistochemical staining

TNF-α, IL-1β, and iNOS were evaluated by immunohistochemistry to assess inflammatory responses in liver tissue. The staining intensity was evaluated semi-quantitatively by a blinded pathologist. Liver tissues from each group were fixed in neutral buffered formalin, embedded in paraffin, and processed according to the manufacturer’s protocol using specific primary antibodies followed by appropriate secondary antibodies. The immunohistochemical staining was visualized using 3,3’-diaminobenzidine (DAB) as a chromogen, and the sections were counterstained with hematoxylin.

### Statistical analysis

Data were analyzed by the Statistical Package for Social Science (SPSS) software, version 18.0 for Windows. Quantitative data were expressed using mean and standard deviation and analyzed using F-test (ANOVA) to compare the different studied groups. Post-hoc Tukey’s test was applied for multiple comparisons between groups when the ANOVA test showed significant differences. P < 0.05 was considered statistically significant for all analyses. All graphs and statistical analyses were performed using GraphPad Prism software (version 8.0).

### Ethical considerations

All experimental procedures and animal handling protocols were reviewed and approved by the Animal Care and Use Committee (ACUC) of Mansoura University, Egypt (Approval Code: MU-ACUC [MED.R.24.09.44]; Approval Date: 15 September 2024), in accordance with institutional and national guidelines for the care and use of laboratory animals. The number of animals used was kept to the minimum needed to get significantly valid results. All efforts were made to minimize animal suffering during the experimental procedures. Animals were monitored daily for any signs of distress, and appropriate measures were taken to ensure their welfare throughout the study period.

## Data Availability

All data supporting the findings of this study are available within the article.
